# Predicting Subclinical Ketosis in Dairy Cows Using Machine Learning Techniques

**DOI:** 10.3390/ani11072131

**Published:** 2021-07-19

**Authors:** Alicja Satoła, Edyta Agnieszka Bauer

**Affiliations:** 1 Department of Genetics, Animal Breeding and Ethology, Faculty of Animal Science, University of Agriculture in Krakow, al. Mickiewicza 24/28, 30-059 Krakow, Poland; 2 Department of Animal Reproduction, Anatomy and Genomics, Faculty of Animal Science, University of Agriculture in Krakow, al. Mickiewicza 24/28, 30-059 Krakow, Poland; edyta.bauer@urk.edu.pl

**Keywords:** hyperketonemia, β-hydroxybutyrate, acetone, metabolic disorder, logistic regression

## Abstract

**Simple Summary:**

The maintenance of cows in good health and physical condition is an important component of dairy cattle management. One of the major metabolic disorders in dairy cows is subclinical ketosis. Due to financial and organizational reasons it is often impossible to test all cows in a herd for ketosis using standard blood examination method. Using milk data from test-day records, obtained without additional costs for breeders, we found diagnostic models identifying cows-at-risk of subclinical ketosis. In addition, to select the best models, we present a general scoring approach for various machine learning models. With our models, breeders can identify dairy cows-at-risk of subclinical ketosis and implement appropriate management strategies and prevent losses in milk production.

**Abstract:**

The diagnosis of subclinical ketosis in dairy cows based on blood ketone bodies is a challenging and costly procedure. Scientists are searching for tools based on results of milk performance assessment that would allow monitoring the risk of subclinical ketosis. The objective of the study was (1) to design a scoring system that would allow choosing the best machine learning models for the identification of cows-at-risk of subclinical ketosis, (2) to select the best performing models, and (3) to validate them using a testing dataset containing unseen data. The scoring system was developed using two machine learning modeling pipelines, one for regression and one for classification. As part of the system, different feature selections, outlier detection, data scaling and oversampling methods were used. Various linear and non-linear models were fit using training datasets and evaluated on holdout, testing the datasets. For the assessment of suitability of individual models for predicting subclinical ketosis, three β-hydroxybutyrate concentration in blood (bBHB) thresholds were defined: 1.0, 1.2 and 1.4 mmol/L. Considering the thresholds of 1.2 and 1.4, the logistic regression model was found to be the best fitted model, which included independent variables such as fat-to-protein ratio, acetone and β-hydroxybutyrate concentrations in milk, lactose percentage, lactation number and days in milk. In the cross-validation, this model showed an average sensitivity of 0.74 or 0.75 and specificity of 0.76 or 0.78, at the pre-defined bBHB threshold 1.2 or 1.4 mmol/L, respectively. The values of these metrics were also similar in the external validation on the testing dataset (0.72 or 0.74 for sensitivity and 0.80 or 0.81 for specificity). For the bBHB threshold at 1.0 mmol/L, the best classification model was the model based on the SVC (Support Vector Classification) machine learning method, for which the sensitivity in the cross-validation was 0.74 and the specificity was 0.73. These metrics had lower values for the testing dataset (0.57 and 0.72 respectively). Regression models were characterized by poor fitness to data (R^2^ < 0.4). The study results suggest that the prediction of subclinical ketosis based on data from test-day records using classification methods and machine learning algorithms can be a useful tool for monitoring the incidence of this metabolic disorder in dairy cattle herds.

## 1. Introduction

Subclinical ketosis is one of the major metabolic disorders in dairy cows [1,2,3]. Subclinical ketosis increases the risk of clinical ketosis [4] as well as other disorders, e.g., displaced abomasum, metritis and lameness [5,6,7], which can lead to an increased culling rate [4] and higher costs at herd level [8,9]. It has been determined that subclinical ketosis is also associated with reduced milk production [6] as well as with reduced reproductive performance of cows [10,11]. Subclinical ketosis is mostly observed during early lactation and can be diagnosed based on an elevated ketone bodies in body fluids (blood, milk, urine) in the absence of clinical signs [12]. The β-hydroxybutyrate concentration in blood (bBHB) is an indicator used for diagnosing subclinical ketosis in dairy cows. The review paper by Benedet et al. [13] indicates various bBHB thresholds used in the literature for distinguishing between healthy cows and those with subclinical ketosis. In the majority of publications, the threshold is defined as 1.2 mmol/L [7,14,15,16,17,18], and only rarely it is defined as 1.0 mmol/L [19,20] or 1.4 mmol/L [6,21]. The detection of ketone bodies in blood is not a standard procedure used in the management of dairy cattle herds. Due to practical (financial and organizational) limitations, it is impossible to test all cows in a herd at regular intervals. A search for indicators in milk samples during the assessment of milk performance, which would allow identifying cows-at-risk of subclinical ketosis during early lactation is ongoing. The strong correlation between the ketone bodies in blood and milk [22] can be an indication for the use of acetone (ACE) and β-hydroxybutyrate concentrations in milk (mBHB) for diagnosing subclinical ketosis. van Knegsel et al. [23] found that the inclusion of ACE and mBHB helps to detect subclinical ketosis with greater accuracy as compared to the inclusion of fat-to-protein ratio in milk.

The incidence of ketosis varies greatly between individual farms. Clinical ketosis is observed in about 4–10% of cows per herd and subclinical ketosis—in about 10–50% of cows [4,15,24]. In Poland, about 10% of cows per herd are at risk of ketosis on average. This percentage is even higher, up to 30%, during the early period of the first lactation [25]. According to Oetzel [1], the identification of subclinical ketosis in 10% of cows in a herd should be considered an alarming level.

The objective of the study was (1) to design a scoring system that would allow choosing the best machine learning models for the identification of cows-at-risk of subclinical ketosis, (2) to evaluate various machine learning methods and to choose the best performing models, and (3) to validate the best performing models using a testing dataset containing unseen data.

The advantages of machine learning methods include the possibility of generating models without any previous knowledge of relationships between variables [26], the smaller number of assumptions concerning data (e.g., normal distribution is often not required), as compared to linear methods [27].

Machine learning has been used in the field of dairy science for early detection of subclinical mastitis [28,29,30,31]. Much attention has been paid to the development of machine learning expert systems for detection of subclinical mastitis from milking parameters. Such parameters as milk yield, fat, protein and lactose concentration, milking time and peak flow are easily accessible due to widely used in dairy farms automatic milking systems, which provide breeders a large amount of information about each cow. Using machine learning techniques and information from non-invasive sensors allow prediction of time-to-calving in beef and dairy cows [32], modeling of milk yield of dairy cows under heat stress condition [33], and identification of heat-stressed cows [34].

In the traditional approach, models are often built as a result of a good understanding of the application domain which helps to create and select variables that can be included in models. Model validation is based mainly on the goodness-of-fit evaluation and hypothesis testing. In machine learning, the effort is shifted from a deep understanding of the application domain towards computationally constructed and tested models [35].

## 2. Materials and Methods

### 2.1. Initial Dataset

The original dataset consisted of 882 test-day (TD) records for Polish Holstein–Friesian cows. Some records were excluded from further analysis if the lactation number was unknown (*n* = 5), the sample collection day was incorrect (<6 or >60 days in milk) (*n* = 37) and the test-day results were missing (*n* = 7). Following the removal, the initial dataset consisted of 833 unique TD records from the first eight lactations, grouped into four categories of lactation (1, 2, 3, 4–8). The cows calved in 37 herds in 2013 and 2014. The data were provided by the Polish Federation of Cattle Breeders and Dairy Farmers. The records included nine milk traits: TD milk yield, fat, protein and lactose percentages, fat-to-protein ratio (FPR), milk urea concentration, somatic cell count (SCC), ACE and mBHB. The daily FPR was calculated as the ratio of TD fat percentage to protein percentage. To normalize the distribution, the SCC in milk was common log-transformed to the somatic cell score (SCS). All milk variables were recorded as continuous traits and were not assigned to categories. The number of lactation was used as a categorical variable. Table 1 shows the descriptive statistics of the initial dataset.

There was only one sample per cow in the dataset. Milk samples were analyzed using a MilkoScan FT6000 analyzer (Foss, Hillerod, Denmark). The acetone and β-hydroxybutyrate concentrations in milk were determined by Fourier-transform infrared method (FTIR) using a CombiFoss analyzer (Foss, Hillerod, Denmark). Sampling of individual cows comprised single milk and blood samples collected on the same test-day. The β-hydroxybutyrate concentrations in blood were measured using an OptiumXido glucometer (Abbott, Winey, UK). The data were collected between September 2013 and June 2014. For further analysis, three bBHB thresholds were used as the diagnostic reference for subclinical ketosis: 1.0, 1.2 and 1.4 mmol/L. Cows with circulating bBHB lower than the pre-defined threshold were considered to be healthy.

### 2.2. Approach

The scoring system for the identification of subclinical ketosis was developed using two machine learning (ML) modeling pipelines, one for regression and one for classification.

**Figure 1:**
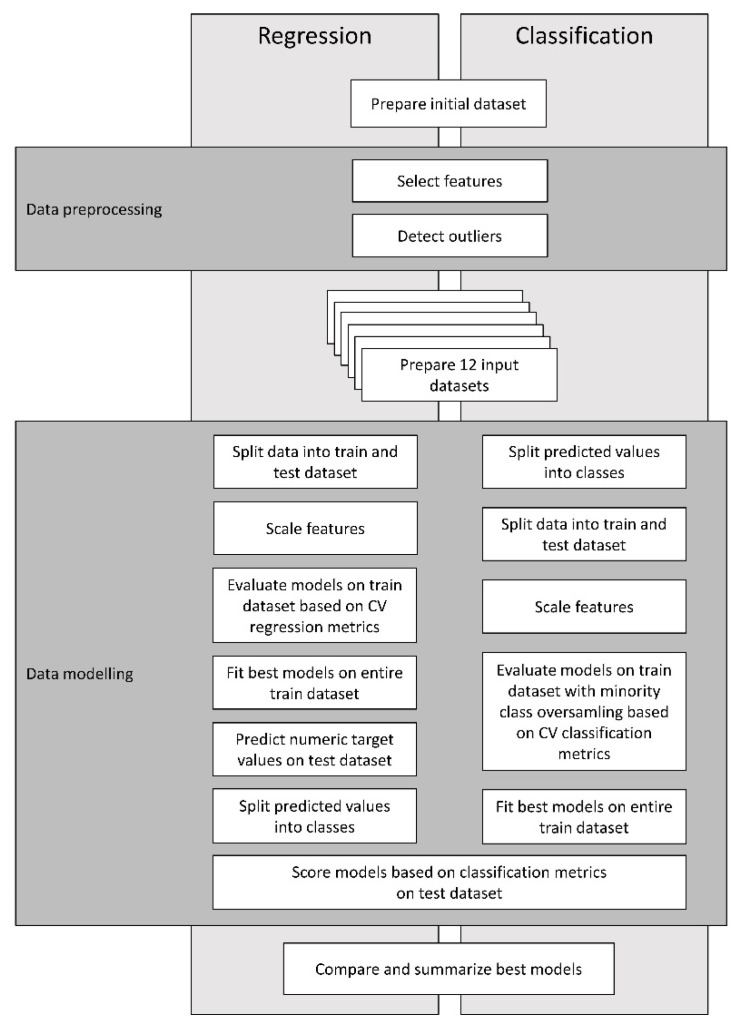
Regression and classification modeling pipelines using cross-validation (CV) for models fitting.

The analyses were performed with Python version 3.8, using pandas (1.1.2), numpy (1.19.2), scipy (1.5.2), imbalanced-learn (0.7.0), scikit-learn (0.23.2), lightgbm (3.0.0), xgboost (1.2.0) and catboost (0.24.2) libraries. Figure 1 presents an overview of the regression and classification modeling pipelines.

### 2.3. Data Pre-Processing for Machine Learning

For the best performance of ML algorithms, 12 versions of the initial dataset were prepared using different feature selection and outlier detection methods.

#### 2.3.1. Feature Selection

To select features for modeling, two feature selection methods were used: one based on Pearson’s and Spearman’s correlation coefficients, and another one based on ML recursive feature elimination method (RFE).

Table 2 shows Pearson’s correlation coefficients for continuous features in the initial dataset. The correlation coefficients between independent features ranged between 0.41 and 0.86. To eliminate multicollinearity between independent features, the ones with correlation coefficient above 0.80 were examined. Fat percentage was eliminated from the modeling dataset because it was highly correlated with FPR (0.86). The acetone and β-hydroxybutyrate concentrations in milk were also highly correlated (0.76), however, below the pre-defined threshold of 0.80. Finally, features correlated with the target variable (bBHB), having an absolute value of correlation coefficient equal to or greater than 0.20, were selected for further processing: ACE (0.63), mBHB (0.62), FPR (0.44), lactose percentage (0.24) and days in milk (DIM) (0.21). In addition, the only categorical feature in the initial dataset (parity) was selected as having Spearman’s correlation coefficient with the target variable of 0.20.

Based on scatter plots of all combinations of features, no non-linear relationships were observed, neither between independent features nor between independent features and the target feature.

The recursive feature elimination machine learning method with scikit-learn DecisionTreeRegressor estimator was used for selecting the three best-suited groups of features. The goal of the recursive feature elimination is to select features by recursively considering smaller and smaller sets of features using an external estimator that assigns weights to features. First, the estimator is trained using the initial set of features to determine the importance of each feature. Then, the least important features are pruned one by one out of the current set of features. That procedure is recursively repeated until the desired number of features is achieved.

The best three groups of features selected using the RFE selection method were termed as RFE1, RFE2, and RFE3. The RFE1 group included ACE only. The RFE2 group contained milk yield, fat percentage, protein percentage, FPR and ACE. The RFE3 group contained protein percentage and ACE.

#### 2.3.2. Outliers

Two approaches: analytical and numerical were used for the identification of outliers. In the analytical approach, for features with non-Gaussian distribution (ACE, mBHB and bBHB), observations with values higher than 1.5 of interquartile range (IQR) were removed. For features with Gaussian distribution (milk yield, FPR, fat, protein and lactose percentages), observations with values higher than three standard deviations (SD) from the mean were removed. In the numerical approach, outliers were detected using unsupervised one-class classification (OOC) approach based on the scikit-learn local outlier factor (LOF) machine learning method. The unsupervised anomaly detection LOF algorithm is a method which computes the local density deviation of a given data point with respect to its neighbors. It considers as outliers the samples that have a substantially lower density than their neighbors. Table 3 summarizes the differences between the 12 datasets generated using different feature selection and outlier detection methods.

### 2.4. Modeling Pipelines—Description and Validation of Models

In order to predict subclinical ketosis based on a numerical (continuous) target feature (bBHB), two ML modeling pipelines were designed and used to score regression and classification models. All the 12 datasets prepared during the data pre-processing stage were used as input for both pipelines.

Each input dataset was split into training (with 70% of observations) and testing (30% of observations) subsets using the scikit-learn *train_test_split* method with the same *random_state* parameter for reproducibility and comparability. Using the same random state guarantees the same split into training and testing datasets at all times. In addition, the stratified sampling method was used in the classification pipeline. The use of such sampling leads to the generation of training and testing subsets that have the same proportions of class labels as in the initial dataset. The same random state and stratified sampling defined while splitting data into the training and testing subsets made it possible to compare different ML algorithms based on the same input data.

Each training dataset was scaled using four scikit-learn feature scaling methods: StandardScaler, RobustScaler, Normalizer and MinMaxScaler. Non-scaled version was also used for comparison. Some algorithms perform better if features are in the same scale or are scaled using a different feature scaling method.

In both pipelines, dummy estimators were used to establish the performance baseline (point of reference) for all other modeling techniques. If a model achieves performance at or below the baseline, the technique should be improved or abandoned.

#### 2.4.1. Regression Pipeline

In the regression pipeline, 14 ML algorithms were used. In the scikit-learn package: DummyRegressor (always returns mean), LinearRegression, ElasticNet, SupportVectorRegressor (SVR) with linear and squared exponential (rbf) kernels, DecisionTreeRegressor, AdaBoostRegressor, BaggingRegressor, RandomForestRegressor, ExtraTreesRegressor, and BayesianRidge; in the xgboost package: XGBRegresor; and in the lightgbm package: LGBMRegressor. All the methods (except SVR) were used with their default hyperparameters. The mathematical details and the conceptual underpinnings of the methods used in the pipelines can be found in Hastie et al. [36].

For each feature scaling method, performance of the fitted models was evaluated using the training dataset by repeating ten times the 10-folds cross validation (CV) with the mean coefficient of determination (R^2^), mean absolute error (MAE), root mean square error (RMSE) and their standard deviations as model performance metrics. Next, the best performing models were fitted to the entire training dataset for making predictions at a later stage (using unseen data represented by the testing dataset).

The testing dataset was used for 12 best regression models (one per dataset) to compare their performance with classification models. bBHB predicted by regression models were split into binary classes based on three cut-off points (1.0, 1.2 and 1.4) and their classification power was evaluated based on sensitivity, specificity, balanced accuracy, Matthews correlation coefficient and F_2_ score classification metrics. bBHB values lower than the cut-off point were classified into ketosis negative class (class label = 0). bBHB values equal to or greater than the cut-off point were classified into ketosis positive class (class label = 1).

#### 2.4.2. Classification Pipeline

The first step was to create three binary target features based on bBHB original continuous values according to three cut-off points at 1.0, 1.2 and 1.4. bBHB values lower than the cut-off point were classified into ketosis negative class (class label = 0). bBHB values equal to or greater than the cut-off point were classified into ketosis positive class (class label = 1).

In the classification pipeline, 12 machine learning algorithms were used. In the scikit-learn package: DummyClassifier, LogisticRegression, SGDClassifier, DecisionTreeClassifier, KNeighborsClassifier, AdaBoostClassifier, BaggingClassifier, RandomForestClassifier, ExtraTreesClassifier, SupportVectorClassification (SVC), and GaussianNB; in the catboost package: CatboostClassifier. For all the methods, their default hyperparameters were used.

Table 4 shows the number of observations in ketosis positive and ketosis negative classes, including the prevalence of subclinical ketosis for each cut-off point of bBHB (1.0, 1.2 and 1.4).

As observed, positive and negative classes were imbalanced in all cases (Table 4). To balance the target binary classes, 5 oversampling methods (from the scikit-learn package) were used: SMOTE, BorderlineSMOTE, RandomOverSampler, ADASYN and SVMSMOTE. The oversampling was performed during the cross-validation iterations after the training dataset was split into folds to eliminate potential data leakage.

For each of the cut-off points, feature scaling and oversampling algorithm classification models were evaluated on the training datasets using the scikit-learn RepeatedStratifiedKFold cross-validation method (repeated ten times with 10-folds). The mean cross-validation sensitivity, specificity, balanced accuracy, Matthews correlation coefficient and F_2_ score, and their standard deviations were used as model performance metrics. Next, the 12 best performing models (one per dataset) were fitted to the entire training dataset for making predictions at a later stage (using unseen data represented by the testing dataset).

Using the testing datasets, for each of the class cut-off points, the 12 best classification models were evaluated based on sensitivity, specificity, balanced accuracy, Matthews correlation coefficient and F_2_ score classification metrics.

The application of different outlier detection methods (during data pre-processing) resulted in a varying number of observations and features in each of the 12 input datasets. The ML algorithms were scored separately for each of the twelve input datasets to ensure that the scoring was performed on the same training and testing datasets for each algorithm in the regression and classification pipeline. As a result, 72 best performing models were selected (one per each input dataset, pipeline and cut-off point).

#### 2.4.3. Evaluation Metrics

To compare and assess the final performance of each regression and classification model on the testing dataset, the continuous values of the target feature (bBHB), as predicted by a regression model, were translated into classes using three cut-off points (1.0, 1.2 and 1.4) and the same logic of positive class assignment as in case of classification. Next, the same set of classification metrics was used consistently across all models, regardless of the initially used type of machine learning method.

Five metrics were used for the evaluation of classification models: sensitivity (recall, true positive rate, TPR), specificity (true negative rate, TNR), balanced accuracy (bACC), Matthews correlation coefficient (MCC) and F_2_ score.

Sensitivity indicated the proportion of cows with subclinical ketosis that were correctly predicted as cows with subclinical ketosis and specificity indicated the proportion of healthy cows that were correctly predicted as healthy.

Instead of accuracy which indicates the percentage of correctly predicted cows in the dataset but can be misleading in the case of an imbalanced dataset, balanced accuracy [37] was calculated using the following formula:

$$\mathrm{bACC}=\frac{\mathrm{sensitivity}+\mathrm{specificity}}{2}$$

The value of this metric can range from 0 to 1, where 1 means perfect performance of a model and 0 means random scoring.

Additionally, the Matthews correlation coefficient [38] was calculated. This metric has values in the range of −1 to 1, where −1 represents the total disagreement between predicted and actual value, and 1 indicates that the prediction generated by the model entirely agrees with the actual value. The MCC was calculated according to the following formula:

$$\mathrm{MCC}=\frac{\mathrm{TP}\cdot \mathrm{TN}-\mathrm{FP}\cdot \mathrm{FN}}{\sqrt{\left(\mathrm{TP}+\mathrm{FP}\right)\left(\mathrm{TP}+\mathrm{FN}\right)\left(\mathrm{TN}+\mathrm{FP}\right)\left(\mathrm{TN}+\mathrm{FN}\right)}}$$

where TP, TN, FP and FN are true positive, true negative, false positive, and false negative, respectively.

The F_β_ score can be interpreted as a weighted harmonic mean of precision and recall reaching the optimal value at 1 (indicating perfect precision and recall) and the least optimal value at 0. Precision is the percent of correct positive predictions. Recall (also referred to as sensitivity) is the percentage of correctly classified positive values.

$$F_{\beta }=\left(1+\beta^{2}\right)\cdot \frac{\mathrm{precision}\cdot \mathrm{recall}}{\beta^{2}\cdot \mathrm{precision}+\mathrm{recall}}$$

The two values commonly used for β are 2, which means that greater weight is attributed to recall than to precision, and 0.5, which means that greater weight is attributed to precision than to recall. For ketosis predictions, it is more important to identify as many cows with subclinical ketosis as possible, and therefore the F_2_ score was chosen as a metric.

$$F_{2}=5\cdot \frac{\mathrm{precision}\cdot \mathrm{recall}}{4\cdot \mathrm{precision}+\mathrm{recall}}$$

## 3. Results

### 3.1. Number of Models

For each of the 12 input datasets (Table 3), characterized by the different compositions of features and the use of different methods for their selection and the elimination of outliers, five datasets were generated, for which different methods were used for the scaling of independent variables (no scaling was performed for one of these five datasets). As a result, a total of 60 sets were generated.

The classification approach included the generation of three sets for each of these 60 sets, where the continuous values of the dependent variable (bBHB) were assigned to the following classes: 0 and 1, based on one of the bBHB thresholds (1.0, 1.2, 1.4). Next, using five oversampling methods, for each of the resulting 180 datasets and 12 machine learning methods, a total of 10800 models were trained.

In the regression-based approach for each of 60 datasets characterized by the different composition of features, the use of different methods for the elimination of outliers and scaling of variables, a total of 840 models were trained using 14 machine learning algorithms.

### 3.2. Performance of Classification Models

In the classification-based approach, out of the models trained for each threshold (1.0, 1.2, 1.4) for the dependent variable (bBHB), a total of 12 models were chosen (one per each dataset shown in Table 3), for which the greatest mean sensitivity, specificity, bACC and MCC were obtained in the cross-validation, including the lowest respective standard deviations. The values of the selected metrics for the best classification models are shown in (Table 5, Table 6 and Table 7).

For the threshold of 1.0 mmol/L for the dependent variable (bBHB), the mean sensitivity in the cross-validation ranged between 0.63 and 0.90 (with standard deviation in the range of 0.09 and 0.20) and the specificity was in the range of 0.14 and 0.73 (with standard deviation in the range of 0.05 and 0.19) (Table 5). The model with both high average sensitivity (0.74) and specificity (0.73) was a model based on the SVC machine learning algorithm. Variables for the model were selected based on the calculated coefficients of correlation between variables and outliers were eliminated using the LOF machine learning method (Table 5, dataset 3). For scaling of variables, RobustScaler was used. Oversampling was performed using the ADASYN method. For this model, bACC obtained in the cross-validation was equal to 0.74. This model was characterized by the highest MCC (0.40) among the models selected for each of the 12 datasets (Table 5). Taking also into account the average F_2_ score (0.63) as determined during the cross-validation, this model should be considered to be superior as compared to the other. For the testing dataset containing unseen data, the sensitivity, specificity and bACC were lower than those obtained in the cross-validation at 0.57, 0.72 and 0.65, respectively.

If the bBHB threshold was defined as 1.2 mmol/L, then the best classification model was the logistic regression (Table 6). Oversampling was performed using the ADASYN method. As previously, variables to be included in the model were selected based on correlation coefficients and they were scaled using the MinMaxScaler (Table 6, datasets 1 and 3). In principle, it was not important whether outliers were eliminated using a machine learning method or were not eliminated because mean values of metrics selected in the cross-validation were similar in both cases: 0.74 and 0.73 for sensitivity, 0.76 and 0.77 for specificity, and bACC was 0.75 for both models. MCC was 0.38 and 0.39 respectively for the model used for a dataset from which outliers were eliminated and for a model used for a dataset where outliers were not eliminated. For the bBHB threshold defined as 1.2 mmol/L, the F_2_ score was highest for the two models indicated by us and it was 0.6 in both cases. For both models, the values of the selected metrics for the testing dataset were close to the mean values of these metrics as obtained in the cross-validation. Taking into account the bBHB threshold of 1.2 mmol/L out of the best performing models (with the greatest sensitivity), each of the 12 datasets also included models for which the average sensitivity obtained in the cross-validation was above 0.8, however, the specificity for these models ranged between 0.14 and 0.30 (Table 6).

For the bBHB threshold of 1.4 mmol/L, the best performing model with high average sensitivity (0.75) and specificity (0.78) obtained in the cross-validation was again a model based on logistic regression (Table 7). As previously, variables to be included in the model were selected based on correlation coefficients and they were scaled using the StandardScaler, and oversampling was performed using the ADASYN method (Table 7, dataset 3). Outliers were removed using the LOF machine learning method. The model based on the SVC machine learning algorithm seems to be an equally good model (cross-validation sensitivity of 0.74 and specificity of 0.79). Calculations for this model were performed based on data with outliers removed using the LOF machine learning method and variables selected using the RFE machine learning method (Table 7, dataset 12). This model included such independent variables as protein percentage and acetone concentration. The variables were scaled using RobustScaler and oversampling was also performed using the ADASYN method. The mean MCC obtained in the cross-validation for the two discussed models were the same (0.38) and highest among the considered models for the bBHB threshold of 1.4 mmol/L (Table 7). The F_2_ score was 0.58 in both cases, which demonstrates the superiority of the models referred to above as compared to the others. The values of the analyzed metrics for the testing dataset were no different from those obtained during the cross-validation (Table 7).

For the best performing models, independent variables were selected based on the calculated coefficients of correlation between variables. The features that were taken into account included FPR, ACE, mBHB, lactose percentage, lactation number and DIM. Oversampling using the ADASYN method was performed for the sets used for fitting of these models. The most desired values of metrics (sensitivity, specificity, bACC, MCC, F_2_ score) were obtained for a logistic regression model (bBHB cut-offs 1.2 and 1.4) as well as for a model based on the SVC (SupportVectorClassification) machine learning algorithm (bBHB cut-off 1.0).

### 3.3. Performance of Regression Models

In the regression-based approach, a total of 12 models were selected out of the trained models (one per each dataset specified in Table 3), for which the greatest R^2^, the lowest MAE and the lowest RMSE were obtained in the cross-validation, having the lowest respective standard deviations. The values of the metrics selected in the cross-validation for the best performing prediction models, for each of the 12 datasets, are shown in Table 8.

The highest R^2^ (0.39) in the cross-validation was obtained for the model based on the SVR machine learning algorithm. For this model, the related features were selected taking into account the values of correlation coefficients (Table 8, dataset 1). No outliers were removed from the set. Variables were scaled using StandardScaler. The MAE for the model in question was 0.34 and the RMSE was 0.55 while for the other models, the MAE was in the range of 0.30 and 0.35, and the RMSE—in the range of 0.44 and 0.58. The low R^2^ score obtained in the cross-validation can be indicative of the limited possibilities for using the regression model for predicting bBHB.

To compare the classification and regression models, the estimated continuous values of the dependent variable bBHB were assigned to two classes (0 and 1) using the same rules that were used for the classification models, taking into account three bBHB thresholds (1.0, 1.2, 1.4). Subsequently, the same metrics were calculated for the testing dataset as those calculated for classification models (Table 9). For the regression-based model characterized by the greatest coefficient R^2^ (0.39) in the cross-validation, the sensitivity for the testing dataset was in the range of 0.32 and 0.40 according to the pre-defined bBHB threshold and the specificity was in the range of 0.94 and 0.97 (Table 9, dataset 1). The sensitivity was lower as compared to the recommended classification models, for which the sensitivity was in the range of 0.57 and 0.74 for the testing dataset.

## 4. Discussion

### 4.1. The Use of Classification Models for Diagnosing Subclinical Ketosis

The classification models most commonly used for diagnosing of cows-at-risk of subclinical ketosis are those based on logistic regression [15,18,39]. In our study, the logistic regression model also proved to be the best, both when the bBHB threshold was defined as 1.2 or 1.4 mmol/L (Table 6 and Table 7, dataset 3). Taking into account the threshold of 1.0 mmol/L, the best performing classification model was the model based on the SVC machine learning algorithm (Table 5, dataset 3). The average sensitivity achieved in our study in the cross-validation for the best performing models ranged between 0.74 and 0.75 (Table 5, Table 6 and Table 7). Chandler et al. [18], who also used a logistic regression model, obtained lower sensitivity of 0.56 and 0.32 for primiparous and multiparous Holstein cows, respectively, and 0.40 and 0.42 for primiparous and multiparous Jersey cows, respectively. On the other hand, van der Drift et al. [15] obtained higher sensitivity (0.82) with equally high specificity (0.84), however, they did not perform cross-validation or external validation for the final model. The specificity obtained in this study (0.73–0.79) for the best models is not as high as that shown in the study of van der Drift et al. [15] and Chandler et al. [18] (0.83–0.99), however, given that the models proposed in this study are characterized by sensitivity, which is quite high, they can be considered for practical use. Denis-Robichaud et al. [40] achieved very high sensitivity and specificity (>0.90) for their model which included only ACE and mBHB, however, the level of ketone bodies in milk was determined using flow-injection analysis and not the FTIR method. The ketone bodies in milk as determined using flow-injection analysis are more strongly correlated with the ketone bodies in blood than the ketone bodies determined using the FTIR method [40].

It should be highlighted that the values of metrics for the best models in the external validation on a testing dataset (about 0.70 for sensitivity and about 0.80 for specificity) were similar to those obtained in the cross-validation, which may be indicative of their good suitability for correct classification of new data.

The most desirable values of metrics were obtained for logistic regression models (or models based on the SVC algorithm) when they were validated using a dataset containing features selected based on correlation coefficients. These features included milk yield parameters such as FPR, ACE, mBHB, and lactose percentage. Additional features accounted for in the models included DIM and lactation number. Less optimal results were obtained for the three datasets containing features selected using the RFE machine learning method. One of these datasets included only ACE, the other one also included protein percentage, and the third one also included—in addition to ACE and protein percentage—milk yield, fat percentage and FPR.

Other authors also included ACE and mBHB in their logistic regression models. Chandler et al. [18], for example, used ACE in all the models studied by them (for primiparous and multiparous Holstein and Jersey cows), however, mBHB was not included in models oriented towards primiparous cows. On the other hand, Denis-Robichaud et al. [40] who took into account only ACE and mBHB, generated a model that allowed predicting subclinical ketosis with sensitivity and specificity greater than 0.90, however, as it was mentioned above, ketone bodies in milk were determined based on flow-injection analysis and not the FTIR method.

The fat-to-protein ratio, in addition to ketone bodies level in milk, was a traditional tool used for screening for ketosis [15,41,42]. Hyperketonemia is associated with an increase in fat percentage and a decrease in protein percentage in milk, which increases the FPR. However, the inclusion by some authors of fat-to-protein ratio as the only feature in a model for predicting subclinical ketosis was not sufficient because the sensitivity of such models was in the range of 0.58 and 0.69 and the specificity—in the range of 0.66 and 0.71 [23,40,41], and these values were lower than those presented in this study.

In future, it would be advisable to extend models for the prediction of subclinical ketosis to include other features, e.g., fatty acids in milk [18]. Fatty acids, mobilized from the fatty tissue, are characterized by a high concentration of long-chain fatty acids [14] which are taken up by the mammary gland and secreted in milk fat. Chandler et al. [18] indicated that Jersey cows, which had subclinical ketosis, produced milk with a higher concentration of monounsaturated fatty acids (MUFA) and trans fatty acids, and a lower concentration of short-chain fatty acids as compared to healthy cows.

In our study, the recommended models also included such features as lactation number or DIM. A number of studies demonstrated that the risk of subclinical ketosis increases with lactation number [18,43,44], and therefore it is reasonable to continue using that feature in models. The logistic regression model generated by Chandler et al. [18] for predicting subclinical ketosis in primiparous cows also included the gestation length and the dry period length. The authors highlighted that primiparous cows with hyperketonemia remained pregnant seven days longer as compared to healthy cows. No such correlation was identified for older cows with subclinical ketosis. The authors suggested that the relationship of the features referred to above and the risk of subclinical ketosis should be studied further.

### 4.2. The Use of Regression Models for Diagnosing Subclinical Ketosis

The study also attempted to use a linear regression model for the prediction of bBHB and subsequently, based on the estimated bBHB, for the classification of cows as healthy or ketosis-affected in accordance with the pre-defined bBHB thresholds. However, even for the best model out of the selected ones, the coefficient R^2^ was relatively low (0.39) (Table 8). This model included the same features as the logistic regression model recommended in our study (FPR, ACE, mBHB, lactose percentage, lactation number and DIM) and it was based on the SVR machine learning algorithm (Table 8, dataset 1). Chandler et al. [18] also tested the suitability of linear regression models for predicting bBHB in primiparous and multiparous Holstein and Jersey cows. Regression models were fitted to data covering two periods: 5–11 DIM and 12–20 DIM. The R^2^ coefficient obtained by those authors in the cross-validation was in the range of 0.20 to 0.71 according to period and breed, and the highest values of the coefficient were obtained for primiparous Holsteins. The RMSE of prediction ranged between 0.29 and 0.92, and it was 0.55 for our best model. The R^2^ coefficients obtained in our study demonstrate that there are limited possibilities of using regression models for predicting bBHB and their application for the identification of cows-at-risk of ketosis. In contrary to our study, Chandler et al. [18] obtained higher sensitivity in the cross-validation for linear regression (0.53–0.74) than for logistic regression (0.31–0.55). In our study, the sensitivity obtained in the external validation using a testing dataset for the best regression model based on the SVR machine learning algorithm ranged between 0.32 and 0.40 according to the pre-defined bBHB (Table 9, dataset 1). To compare, the sensitivity for a testing dataset for the recommended classification models ranged between 0.57 and 0.74, and it was higher in the cross-validation (0.73–0.75) (Table 5, Table 6 and Table 7).

## 5. Conclusions

The study evaluated various machine learning algorithms designed for predicting if a cow is at risk of subclinical ketosis. The logistic regression model was found to be the best fitted model, which included features such as fat-to-protein ratio, acetone and β-hydroxybutyrate concentrations in milk, lactose percentage, lactation number and days in milk. Regression models were characterized by poor fitness to data. In the event that it is possible to acquire additional features as determined during the assessment of milk performance (e.g., milk fatty acids), it should be considered including such features in the model and validating the model with the new features. A greater number of observations, including repeated test-day records, could also help to achieve better results using the model. Using machine learning models and milk data, breeders can efficiently identify dairy cows-at-risk of subclinical ketosis and implement appropriate management strategies to optimize or prevent losses in milk production.

## Figures and Tables

**Table 1:** Number of cows, mean and standard deviation of blood β-hydroxybutyrate concentration (bBHB), milk yield, fat percentage, protein percentage, fat-to-protein ratio (FPR), lactose percentage, milk urea concentration (MU), somatic cell score (SCS), acetone and milk β-hydroxybutyrate concentrations (mBHB) according to lactation number.

Item	Lactation 1	Lactation 2	Lactation 3	Lactation ≥ 4
Number of cows	324	202	155	152
bBHB (mmol/L)	0.60 ± 0.45	0.83 ± 0.87	0.93 ± 0.90	0.93 ± 0.87
Milk variables				
Milk (kg)	31.5 ± 7.9	39.2 ± 10.3	39.1 ± 10.5	38.4 ± 10.9
Fat (%)	3.88 ± 0.72	4.14 ± 1.03	4.12 ± 1.00	4.30 ± 0.97
Protein (%)	3.07 ± 0.33	3.12 ± 0.34	3.06 ± 0.35	3.06 ± 0.37
FPR	1.27 ± 0.24	1.33 ± 0.32	1.35 ± 0.32	1.42 ± 0.34
Lactose (%)	4.96 ± 0.20	4.88 ± 0.21	4.85 ± 0.19	4.82 ± 0.23
MU (mg/L)	198 ± 60	202 ± 71	189 ± 75	177 ± 69
SCS	3.37 ± 1.93	2.86 ± 1.88	3.54 ± 2.17	3.80 ± 2.26
Acetone (mmol/L)	0.06 ± 0.09	0.09 ± 0.16	0.09 ± 0.14	0.10 ± 0.15
mBHB (mmol/L)	0.05 ± 0.05	0.07 ± 0.08	0.08 ± 0.08	0.09 ± 0.09

**Table 2:** Pearson’s correlation coefficient for continuous variables in the initial dataset: milk yield, fat percentage, protein percentage, fat-to-protein ratio (FPR), acetone (ACE) and milk β-hydroxybutyrate (mBHB) concentrations, lactose percentage, milk urea concentration (MU), somatic cell score (SCS) and blood β-hydroxybutyrate concentration (bBHB).

Variable	Milk	Fat	Protein	FPR	ACE	mBHB	Lactose	MU	SCS	bBHB
Milk (kg)	1	−0.21	−0.22	−0.12	−0.17	−0.20	0.14	0.06	−0.19	−0.09
Fat (%)		1	0.30	0.86	0.49	0.56	−0.36	0.01	0.14	0.43
Protein (%)			1	−0.22	0.05	−0.04	−0.27	−0.01	0.17	−0.01
FPR				1	0.46	0.59	−0.23	0	0.05	0.44
ACE (mmol/L)					1	0.76	−0.41	−0.05	0.15	0.63
mBHB (mmol/L)						1	−0.40	−0.11	0.16	0.62
Lactose (%)							1	0.05	−0.38	−0.24
MU (mg/L)								1	−0.05	−0.07
SCS									1	0
bBHB (mmol/L)										1

**Table 3:** Characteristics of datasets used for modeling and derived from the initial dataset using feature selection methods, outlier detection methods and independent features included in each dataset (parity, days in milk (DIM), milk yield, fat percentage, protein percentage, fat-to-protein ratio (FPR), lactose percentage, acetone (ACE) and milk β-hydroxybutyrate concentrations (mBHB)).

Dataset Number	Feature Selection Method ^1^	Outlier Detection Method ^2^	Number of Observations	Independent Features Used for Modeling
1	Correlation	none	833	parity, DIM, FPR, ACE, mBHB, lactose
2	Correlation	IQR/SD	783	parity, DIM, FPR, ACE, mBHB, lactose
3	Correlation	LOF	792	parity, DIM, FPR, ACE, mBHB, lactose
4	RFE	none	833	ACE
5	RFE	IQR/SD	776	ACE
6	RFE	LOF	811	ACE
7	RFE	none	833	milk, fat, protein, FPR, ACE
8	RFE	IQR/SD	776	milk, fat, protein, FPR, ACE
9	RFE	LOF	811	milk, fat, protein, FPR, ACE
10	RFE	none	833	protein, ACE
11	RFE	IQR/SD	776	protein, ACE
12	RFE	LOF	811	protein, ACE

^1^ RFE, recursive feature elimination. ^2^ IQR, interquartile range; SD, standard deviation; LOF, local outlier factor.

**Table 4:** Number of cows per input dataset with subclinical ketosis (SCK) and without SCK (no SCK) for each cut-off point (1.0, 1.2, 1.4) of blood β-hydroxybutyrate concentration (bBHB) and the prevalence of SCK positive samples.

Dataset Number	bBHB Cut-Off								
	1.0			1.2			1.4		
	No SCK	SCK	SCK Prevalence (%)	No SCK	SCK	SCK Prevalence (%)	No SCK	SCK	SCK Prevalence (%)
1	670	163	19.6	709	124	14.9	737	96	11.5
2	658	125	16.0	696	87	11.1	721	62	7.9
3	636	156	19.7	673	119	15.0	701	91	11.5
4	670	163	19.6	709	124	14.9	737	96	11.5
5	650	126	16.2	688	88	11.3	713	63	8.1
6	656	155	19.1	695	116	14.3	722	89	11.0
7	670	163	19.6	709	124	14.9	737	96	11.5
8	650	126	16.2	688	88	11.3	713	63	8.1
9	656	155	19.1	695	116	14.3	722	89	11.0
10	670	163	19.6	709	124	14.9	737	96	11.5
11	650	126	16.2	688	88	11.3	713	63	8.1
12	656	155	19.1	695	116	14.3	722	89	11.0

**Table 5:** Sensitivity (TPR), specificity (TNR), balanced accuracy (bACC), Matthews correlation coefficient (MCC) and F_2_ score of the cross-validation on training and testing datasets for models predicting subclinical ketosis (defined as blood β-hydroxybutyrate ≥1.0 mmol/L) in Polish Holstein–Friesian cows.

Dataset Number	Model ^1^	Scaler Method ^2^	Oversampling Method ^3^	Training (Mean ± SD)					Testing				
				Sensitivity (TPR)	Specificity (TNR)	bACC	MCC	F_2_	TPR	TNR	bACC	MCC	F_2_
1	SGD	MMS	BSMOTE	0.72 ± 0.20	0.70 ± 0.19	0.71 ± 0.08	0.37 ± 0.14	0.60 ± 0.13	0.78	0.74	0.76	0.43	0.66
2	LOG	STS	ADASYN	0.66 ± 0.16	0.73 ± 0.07	0.69 ± 0.08	0.30 ± 0.12	0.54 ± 0.11	0.71	0.73	0.72	0.34	0.58
3	SVC	RBS	ADASYN	0.74 ± 0.14	0.73 ± 0.07	0.74 ± 0.07	0.40 ± 0.12	0.63 ± 0.11	0.57	0.72	0.65	0.25	0.50
4	CAT	STS	BSMOTE	0.67 ± 0.14	0.71 ± 0.10	0.69 ± 0.07	0.32 ± 0.12	0.57 ± 0.10	0.57	0.70	0.63	0.22	0.49
5	LOG	NOR	SMOTE	0.90 ± 0.09	0.14 ± 0.05	0.52 ± 0.05	0.04 ± 0.12	0.48 ± 0.05	0.87	0.18	0.52	0.05	0.48
6	LOG	STS	BSMOTE	0.64 ± 0.13	0.73 ± 0.06	0.69 ± 0.08	0.31 ± 0.13	0.55 ± 0.11	0.60	0.75	0.67	0.29	0.53
7	SVC	none	SMOTE	0.74 ± 0.12	0.50 ± 0.08	0.62 ± 0.07	0.20 ± 0.11	0.55 ± 0.08	0.61	0.53	0.57	0.11	0.47
8	SVC	none	BSMOTE	0.78 ± 0.16	0.38 ± 0.10	0.58 ± 0.09	0.12 ± 0.13	0.49 ± 0.09	0.87	0.28	0.57	0.12	0.51
9	SVC	none	SMOTE	0.75 ± 0.15	0.45 ± 0.10	0.60 ± 0.08	0.16 ± 0.12	0.53 ± 0.09	0.74	0.54	0.64	0.22	0.56
10	SVC	STS	BSMOTE	0.75 ± 0.11	0.63 ± 0.07	0.69 ± 0.06	0.31 ± 0.10	0.60 ± 0.08	0.76	0.61	0.68	0.29	0.59
11	SVC	RBS	ADASYN	0.63 ± 0.17	0.66 ± 0.08	0.65 ± 0.09	0.23 ± 0.14	0.49 ± 0.12	0.66	0.65	0.65	0.23	0.51
12	KNN	NOR	ADASYN	0.71 ± 0.14	0.60 ± 0.07	0.65 ± 0.08	0.24 ± 0.12	0.55 ± 0.10	0.66	0.54	0.60	0.16	0.50

^1^ SGD, SGDClassifier; LOG, LogisticRegression; SVC, SupportVectorClassification; CAT, CatBoostClassifier; KNN, KNeighborsClassifier. ^2^ MMS, MinMaxScaler; STS, StandardScaler; RBS, RobustScaler; NOR, Normalizer. ^3^ BSMOTE, BorderlineSMOTE.

**Table 6:** Sensitivity (TPR), specificity (TNR), balanced accuracy (bACC), Matthews correlation coefficient (MCC) and F_2_ score of the cross-validation on training and testing datasets for models predicting subclinical ketosis (defined as blood β-hydroxybutyrate ≥1.2 mmol/L) in Polish Holstein–Friesian cows.

Dataset Number	Model ^1^	Scaler Method ^2^	Oversampling Method ^3^	Training (Mean ± SD)					Testing				
				Sensitivity (TPR)	Specificity (TNR)	bACC	MCC	F_2_	TPR	TNR	bACC	MCC	F_2_
1	LOG	MMS	ADASYN	0.73 ± 0.15	0.77 ± 0.06	0.75 ± 0.08	0.39 ± 0.12	0.60 ± 0.11	0.68	0.80	0.74	0.38	0.58
2	LOG	MMS	ROS	0.65 ± 0.17	0.74 ± 0.06	0.69 ± 0.09	0.27 ± 0.12	0.48 ± 0.12	0.77	0.76	0.76	0.36	0.57
3	LOG	MMS	ADASYN	0.74 ± 0.14	0.76 ± 0.06	0.75 ± 0.07	0.38 ± 0.12	0.60 ± 0.10	0.72	0.80	0.76	0.42	0.62
4	LOG	NOR	SMOTE	0.97 ± 0.06	0.15 ± 0.05	0.56 ± 0.04	0.12 ± 0.07	0.49 ± 0.03	0.86	0.17	0.52	0.03	0.45
5	LOG	NOR	SMOTE	0.90 ± 0.10	0.15 ± 0.05	0.53 ± 0.06	0.05 ± 0.10	0.39 ± 0.04	0.96	0.16	0.56	0.11	0.41
6	LOG	NOR	SMOTE	0.94 ± 0.08	0.14 ± 0.05	0.54 ± 0.04	0.08 ± 0.09	0.47 ± 0.04	0.94	0.19	0.57	0.12	0.48
7	SVC	none	ADASYN	0.77 ± 0.15	0.41 ± 0.09	0.59 ± 0.08	0.13 ± 0.13	0.47 ± 0.09	0.81	0.48	0.64	0.21	0.52
8	SVC	none	ADASYN	0.87 ± 0.14	0.30 ± 0.07	0.58 ± 0.07	0.12 ± 0.10	0.42 ± 0.06	0.69	0.19	0.44	-0.09	0.31
9	SVC	none	ADASYN	0.83 ± 0.15	0.27 ± 0.07	0.55 ± 0.08	0.08 ± 0.13	0.45 ± 0.08	0.91	0.25	0.58	0.14	0.49
10	SGD	MMS	BSMOTE	0.68 ± 0.19	0.73 ± 0.19	0.71 ± 0.09	0.35 ± 0.17	0.55 ± 0.12	0.76	0.69	0.73	0.33	0.58
11	KNN	MMS	ADASYN	0.53 ± 0.18	0.67 ± 0.07	0.60 ± 0.09	0.13 ± 0.13	0.37 ± 0.12	0.54	0.68	0.61	0.15	0.38
12	SGD	STS	BSMOTE	0.66 ± 0.19	0.68 ± 0.17	0.67 ± 0.09	0.27 ± 0.15	0.50 ± 0.13	0.74	0.71	0.73	0.33	0.58

^1^ LOG, LogisticRegression; SVC, SupportVectorClassification; SGD, SGDClassifier; KNN, KNeighborsClassifier. ^2^ MMS, MinMaxScaler; NOR, Normalizer; STS, StandardScaler. ^3^ ROS, RandomOverSampler; BSMOTE, BorderlineSMOTE.

**Table 7:** Sensitivity (TPR), specificity (TNR), balanced accuracy (bACC), Matthews correlation coefficient (MCC) and F_2_ score of the cross-validation on training and testing datasets for models predicting subclinical ketosis (defined as blood β-hydroxybutyrate ≥1.4 mmol/L) in Polish Holstein–Friesian cows.

Dataset Number	Model ^1^	Scaler Method ^2^	Oversampling Method	Training (Mean ± SD)					Testing				
				Sensitivity (TPR)	Specificity (TNR)	bACC	MCC	F_2_	TPR	TNR	bACC	MCC	F_2_
1	SGD	RBS	ADASYN	0.73 ± 0.21	0.71 ± 0.13	0.72 ± 0.10	0.31 ± 0.14	0.52 ± 0.13	0.79	0.67	0.73	0.31	0.55
2	KNN	STS	ADASYN	0.58 ± 0.22	0.77 ± 0.06	0.67 ± 0.11	0.21 ± 0.14	0.39 ± 0.14	0.42	0.80	0.61	0.14	0.31
3	LOG	STS	ADASYN	0.75 ± 0.17	0.78 ± 0.06	0.76 ± 0.08	0.38 ± 0.12	0.58 ± 0.12	0.74	0.81	0.77	0.40	0.59
4	LOG	NOR	SMOTE	0.97 ± 0.07	0.17 ± 0.05	0.57 ± 0.04	0.12 ± 0.07	0.43 ± 0.04	0.97	0.13	0.55	0.10	0.42
5	LOG	NOR	SMOTE	0.98 ± 0.07	0.15 ± 0.05	0.56 ± 0.04	0.10 ± 0.06	0.33 ± 0.03	0.89	0.16	0.53	0.04	0.31
6	LOG	NOR	SMOTE	0.97 ± 0.07	0.15 ± 0.05	0.56 ± 0.04	0.11 ± 0.07	0.41 ± 0.03	0.96	0.17	0.57	0.12	0.41
7	GNB	NOR	ADASYN	0.77 ± 0.18	0.52 ± 0.10	0.65 ± 0.09	0.19 ± 0.11	0.46 ± 0.10	0.83	0.51	0.67	0.22	0.48
8	SVC	none	ADASYN	0.85 ± 0.15	0.33 ± 0.07	0.59 ± 0.08	0.11 ± 0.09	0.34 ± 0.06	0.68	0.38	0.53	0.04	0.29
9	SVC	none	ADASYN	0.79 ± 0.16	0.49 ± 0.08	0.64 ± 0.09	0.18 ± 0.11	0.44 ± 0.09	0.67	0.58	0.62	0.15	0.41
10	KNN	none	ADASYN	0.62 ± 0.20	0.73 ± 0.06	0.67 ± 0.10	0.24 ± 0.14	0.46 ± 0.14	0.66	0.71	0.69	0.25	0.48
11	LOG	STS	SMOTE	0.59 ± 0.23	0.71 ± 0.06	0.65 ± 0.11	0.18 ± 0.13	0.44 ± 0.13	0.79	0.78	0.78	0.35	0.54
12	SVC	RBS	ADASYN	0.74 ± 0.15	0.79 ± 0.07	0.77 ± 0.07	0.38 ± 0.11	0.58 ± 0.10	0.67	0.82	0.74	0.36	0.55

^1^ SGD, SGDClassifier; KNN, KNeighborsClassifier; LOG, LogisticRegression; GNB, GaussianNB; SVC, SupportVectorClassification. ^2^ RBS, RobustScaler; STS, StandardScaler; NOR, Normalizer

**Table 8:** Coefficient of determination (R^2^), mean absolute error (MAE), root mean square error (RMSE) of the cross-validation on training dataset for regression models that predicted blood β-hydroxybutyrate concentration in Polish Holstein–Friesian cows.

Dataset Number	Model ^1^	Scaler Method ^2^	Training (Mean ± SD)		
			R^2^	MAE	RMSE
1	SVR—linear	STS	0.39 ± 0.26	0.34 ± 0.05	0.55 ± 0.12
2	BayesianRidge	none	0.14 ± 0.20	0.30 ± 0.04	0.44 ± 0.10
3	SVR—linear	STS	0.35 ± 0.15	0.35 ± 0.06	0.58 ± 0.15
4	SVR—linear	none	0.37 ± 0.26	0.35 ± 0.05	0.55 ± 0.10
5	BayesianRidge	none	0.08 ± 0.15	0.34 ± 0.05	0.50 ± 0.11
6	SVR—linear	none	0.21 ± 0.29	0.35 ± 0.05	0.56 ± 0.12
7	SVR—linear	none	0.37 ± 0.32	0.34 ± 0.05	0.55 ± 0.11
8	SVR—rbf	MMS	0.17 ± 0.14	0.31 ± 0.05	0.48 ± 0.12
9	SVR—linear	MMS	0.24 ± 0.24	0.34 ± 0.05	0.56 ± 0.13
10	SVR—linear	none	0.36 ± 0.27	0.35 ± 0.05	0.55 ± 0.10
11	BayesianRidge	none	0.08 ± 0.15	0.34 ± 0.05	0.50 ± 0.12
12	SVR—linear	NOR	0.21 ± 0.26	0.35 ± 0.06	0.56 ± 0.13

^1^ SVR—linear, SupportVectorRegressor with linear kernel; SVR—rbf, SupportVectorRegressor with squared exponential kernel. ^2^ STS, StandardScaler; MMS, MinMaxScaler; NOR, Normalizer.

**Table 9:** Sensitivity (TPR), specificity (TNR), balanced accuracy (bACC), Matthews correlation coefficient (MCC) and F_2_ score on testing dataset for regression models that predicted blood β-hydroxybutyrate concentration (bBHB) and diagnosed subclinical ketosis in Polish Holstein–Friesian cows according to three bBHB cut-off points.

Dataset Number	Model ^1^	Scaler Method ^2^	bBHB Cut-Off 1.0					bBHB Cut-Off 1.2					bBHB Cut-Off 1.4				
			TPR	TNR	bACC	MCC	F_2_	TPR	TNR	bACC	MCC	F_2_	TPR	TNR	bACC	MCC	F_2_
1	SVR—linear	STS	0.38	0.94	0.66	0.37	0.40	0.40	0.96	0.68	0.44	0.43	0.32	0.97	0.65	0.40	0.36
2	BayesianRidge	none	0.34	0.90	0.62	0.26	0.35	0.12	0.98	0.55	0.16	0.13	0.16	0.99	0.57	0.28	0.19
3	SVR—linear	STS	0.33	0.96	0.65	0.40	0.37	0.26	0.98	0.62	0.38	0.30	0.23	1.00	0.61	0.42	0.27
4	SVR—linear	none	0.25	0.94	0.60	0.25	0.28	0.26	0.96	0.61	0.30	0.29	0.19	0.98	0.59	0.27	0.22
5	BayesianRidge	none	0.38	0.95	0.66	0.37	0.40	0.20	0.97	0.58	0.20	0.21	0.08	0.99	0.53	0.12	0.10
6	SVR—linear	none	0.32	0.98	0.65	0.44	0.36	0.34	0.99	0.67	0.50	0.39	0.37	1.00	0.68	0.55	0.42
7	SVR—linear	none	0.33	0.94	0.63	0.33	0.36	0.29	0.96	0.62	0.33	0.32	0.23	0.96	0.59	0.26	0.25
8	SVR—rbf	MMS	0.28	0.96	0.62	0.32	0.31	0.20	0.98	0.59	0.24	0.22	0.25	0.98	0.62	0.30	0.27
9	SVR—linear	MMS	0.32	0.97	0.64	0.41	0.36	0.31	0.99	0.65	0.45	0.36	0.30	1.00	0.65	0.52	0.35
10	SVR—linear	none	0.21	0.94	0.57	0.21	0.23	0.26	0.96	0.61	0.30	0.29	0.19	0.98	0.59	0.27	0.22
11	BayesianRidge	none	0.34	0.95	0.65	0.35	0.37	0.20	0.98	0.59	0.24	0.22	0.08	0.99	0.54	0.15	0.10
12	SVR—linear	NOR	0.32	0.98	0.65	0.46	0.36	0.34	1.00	0.67	0.53	0.39	0.33	1.00	0.67	0.55	0.38

^1^ SVR—linear, SupportVectorRegressor with linear kernel; SVR—rbf, SupportVectorRegressor with squared exponential kernel. ^2^ STS, StandardScaler; MMS, MinMaxScaler; NOR, Normalizer.

## Data Availability

The data are publicly unavailable due to data confidentiality.
